# Exploring the Role of *TaPLC1-2B* in Heat Tolerance at Seedling and Adult Stages of Wheat through Transcriptome Analysis

**DOI:** 10.3390/ijms242316583

**Published:** 2023-11-21

**Authors:** Chenyang Li, Ahui Zhao, Yan Yu, Chao Cui, Quan Zeng, Wei Shen, Yang Zhao, Fei Wang, Jian Dong, Xiang Gao, Mingming Yang

**Affiliations:** 1 College of Agronomy, Northwest A&F University, Yangling, Xianyang 712100, China; cyli@nwafu.edu.cn (C.L.); yany@nwafu.edu.cn (Y.Y.); cuichao@nwafu.edu.cn (C.C.); zeng_quan6@163.com (Q.Z.); shenwei1009@126.com (W.S.); zyyyla9233@163.com (Y.Z.); wangfei011128@163.com (F.W.); dj4322@163.com (J.D.); gx@nwsuaf.edu.cn (X.G.); 2 College of Plant Protection, Nanjing Agricultural University, Nanjing 210095, China; ahui96914@163.com

**Keywords:** *Triticum aestivum*, different periods, transcriptome, differentially expressed genes, heat stress

## Abstract

Heat stress is a major abiotic stress that can cause serious losses of a crop. Our previous work identified a gene involved in heat stress tolerance in wheat, *TaPLC1-2B*. To further investigate its mechanisms, in the present study, *TaPLC1-2B* RNAi-silenced transgenic wheat and the wild type were comparatively analyzed at both the seedling and adult stages, with or without heat stress, using transcriptome sequencing. A total of 15,549 differentially expressed genes (DEGs) were identified at the adult stage and 20,535 DEGs were detected at the seedling stage. After heat stress, an enrichment of pathways such as phytohormones and mitogen-activated protein kinase signaling was mainly found in the seedling stage, and pathways related to metabolism, glycerophospholipid metabolism, circadian rhythms, and ABC transporter were enriched in the adult stage. Auxin and abscisic acid were downregulated in the seedling stage and vice versa in the adult stage; and the *MYB*, *WRKY*, and no apical meristem gene families were downregulated in the seedling stage in response to heat stress and upregulated in the adult stage in response to heat stress. This study deepens our understanding of the mechanisms of *TaPLC1-2B* in regard to heat stress in wheat at the seedling and adult stages.

## 1. Introduction

High temperatures are a major stressor affecting crop growth [1]. However, global temperatures are increasing due to factors such as greenhouse gas emissions, and global temperatures are projected to rise by 2–3 °C by the year 2100 [2]. Rising temperatures have a severe negative impact on crop yields, with global crop yields decreasing by as much as 6% for every 1 °C increase in global temperature [3]. Wheat is a cold-loving crop that is grown across more than 220 million hectares globally [4]. As one of the population’s staple crops, it is critical to ensure stable wheat production [5]. The cold-loving nature of wheat results in its sensitivity to high temperatures, and heat stress can cause the plant multiple injuries, the most significant of which being damage to the chloroplast photosynthesis equipment, which affects wheat growth and grain filling [6].

Phospholipase, the most important hydrolase in plants, has been confirmed to be present in various plants, such as *Arabidopsis*, wheat, maize, and rice [7,8,9,10]. Phospholipases are divided into two categories—specific phospholipase C (PI-PLC) and non-specific phospholipase C (PC-NPC)—based on different hydrolytic substrates [10]. The hydrolysis of phosphatidylinositol-4,5-diphosphate (PIP2) in plants to produce inositol-1,4,5-triphosphate (IP3) and diacylglycerol (DAG) is called specific phospholipase C (PLC), while the hydrolysis of other phospholipids—such as phosphatidylcholine (PC)—is called non-specific phospholipase C (NPC) [10,11]. The protein of PLC contains catalytic active X and Y domains, which are necessary for PLC to perform catalytic functions. In addition, the C2 domain at the C-terminus is responsible for phospholipid binding, and the EF motif exists at the N-terminus of the protein. NPCs in plants only exist in the phospholipase domain, which is necessary for their function, and some NPCs have putative signaling peptides [10,12].

It is known that PLCs in plants play roles in growth and development. For example, *Arabidopsis AtPLC2* affects root development by regulating auxin (AUX), and *AtPLC2* mutants show the phenotype of damaged root hairs and shorter roots [13]. Previous studies have also shown that inhibition of PLC activity results in pollen tube growth defects [14]. However, PLCs have additional functions in abiotic stress responses. For example, overexpression of *Arabidopsis AtPLC3*, tobacco *NtPLCδ1*, and maize *ZmPLC1* were shown to increase drought tolerance [15,16,17]. Sagar et al. [11] found that *CaPLC1*, *CaPLC3*, and *CaPLC4* were highly expressed under drought stress. Similarly, overexpression of *AtPLC3* decreased stomatal conductance and reduced water loss, thereby increasing drought tolerance in *Arabidopsis* [18]. There is increasing evidence that PLC is also involved in the salt stress response. *Vigna radiata VrPLC3* was shown to be highly expressed under salt stress, and mutants of *Arabidopsis AtPLC4* showed increased salt tolerance [19,20]. Recently, mutants of rice *OsPLC4* were found to have reduced survival under salt stress, as mutations in *OsPLC4* inhibited the increase in calcium ions and inositol trisphosphate, leading to salt stress sensitivity [21]. In addition to drought and salt stress, PLC has been demonstrated to play a role in heat stress responses. For example, *AtPLC3* and *AtPLC9* take part in heat stress signaling by triggering an increase in calcium ions through an increase in IP3 content under heat stress [22].

Transcriptome sequencing technology (RNA-seq) is an efficient and powerful tool for resolving regulatory mechanisms and mining candidate genes. RNA-seq technology has been widely used in various studies, especially those on abiotic and biotic stresses [23,24]. Azameti et al. [25] conducted RNA-seq after the heat treatment of wheat genotype Raj 3765 and found that metabolic pathways and the synthesis of secondary metabolites were the main pathways of enrichment. They also found that transcription factors (TFs) such as MYB, bHLH, and WRKY were involved in heat stress responses. Saidi et al. [26] used RNA-seq analysis of wheat under salt, drought, heat, and cold stresses to search for differentially expressed genes (DEGs) common to different stresses, and identified 15 stress-responsive candidate genes. Zhang et al. [27] found, by comparing transcriptomics, that genes involved in unfolded protein response (UPR) were specifically enriched with reproductive tissues. Although RNA-seq is widely used in the analysis of heat stress mechanisms in wheat, there is little research on the differences in thermal response mechanisms between the seedling and adult stages of wheat.

Our study discovered that wheat (*Triticum aestivum* L.) with *TaPLC1-2B* RNAi silencing demonstrated greater heat tolerance during the seedling stage. In order to investigate the differences in heat stress response mechanisms between the seedling and adult stages of wheat, heat stress was applied to *TaPLC1-2B* RNAi-silenced KN199 (*PLC*) and wild-type KN199 (WT) wheat varieties during both the seedling and adult stages. Subsequently, the analyzed transcriptomic data were used to reveal the differences in the molecular response mechanisms to heat stress between the seedling and adult stages.

## 2. Results

### 2.1. Processing and Evaluating Transcripts

**Figure 1:**
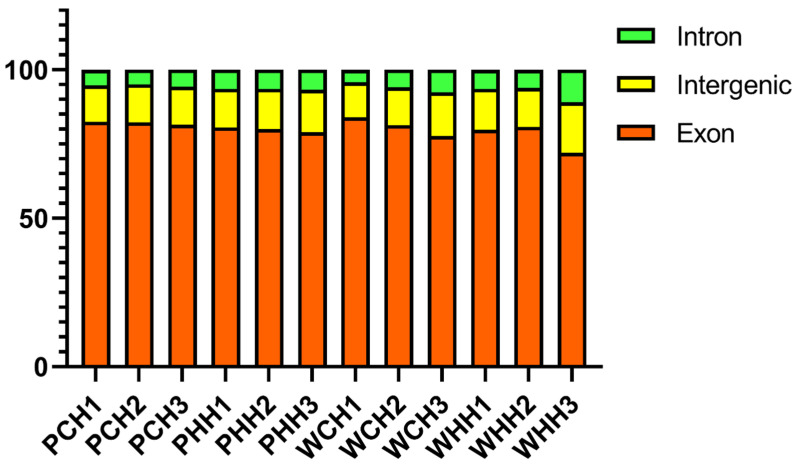
Distribution of reads among different samples during the adult stage. PCH is the untreated *TaPLC1-2B* silenced wheat, PHH is the heat-treated *TaPLC1-2B* silenced wheat, WCH is the untreated WT wheat, and WHH is the treated WT wheat.

For RNA-seq, the following samples were selected: wild-type (WT) before heat treatment (WC) at the seedling stage; WT after heat treatment (WH) at the seedling stage; *PLC* before and after heat treatment at the seedling stage (PC and PH, respectively); WT before and after heat treatment at the adult stage (WCH and WHH, respectively); and *PLC* before and after heat treatment at the adult stage (PCH and PHH, respectively). After removing low-quality reads that contained splices, the total number of high-quality reads during the adult plant stage was 39,038,027. Using IWGSC2.1 (the International Wheat Genome Sequencing Consortium) as the reference genome for sequence alignment and subsequent analysis, the analysis revealed that around 80% of the reads at the adult plant stage were aligned to the exonic region, with 14% aligned to the intergenic region and 6% aligned to the intronic region (Figure 1). The GC content was roughly 54%, and the Q30 base content was more than 94% (Table S1). Based on this data, it was evident that the sequencing was of excellent quality and could be employed for further analysis.

### 2.2. Analysis and Identification of DEGs in Various Time Periods

**Figure 2:**
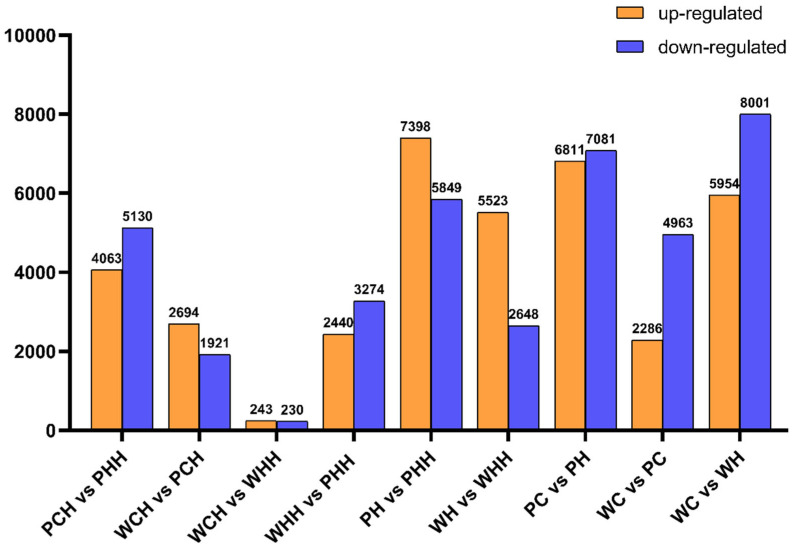
Differential expression genes at different stages. PH is the heat-treated *TaPLC1-2B* silent wheat during the seedling stage, while WH is the treated WT wheat.

By analyzing the RNA-seq data at the adult and seedling stages, with fold change ≥ 2 and FDR < 0.01 as the screening criteria, a total of 15,549 and 20,535 DEGs were identified at the adult and seedling stages, respectively, and 15,149 DEGs were identified during the joint analysis of the seedling and adult stages. PH vs. PHH and WH vs. WHH revealed that the number of upregulated genes was higher than the number of downregulated genes. There was a large difference between the number of upregulated and downregulated genes in WH vs. WHH, which had two times more upregulated genes than downregulated genes, at 5523 and 2648 DEGs, respectively (Figure 2). Interestingly, the number of downregulated genes was higher than the number of upregulated genes after heat treatment at the seedling and adult plant stages. A comparison between the adult stage and the seedling stage after heat stress revealed that the gap between upregulated and downregulated genes was smaller in *PLC* than in WT. After heat stress, the seedling stage WT had more downregulated genes than upregulated genes, at 8001 and 5954 DEGs, respectively (Figure 2). The fact that the number of *PLC* upregulated genes was greater than the number of downregulated genes in the adult stage, while the opposite was observed in the seedling stage, could be because the *TaPLC1-2B* gene specifically regulated the expression of related genes at the adult stage (Figure 2).

**Figure 3:**
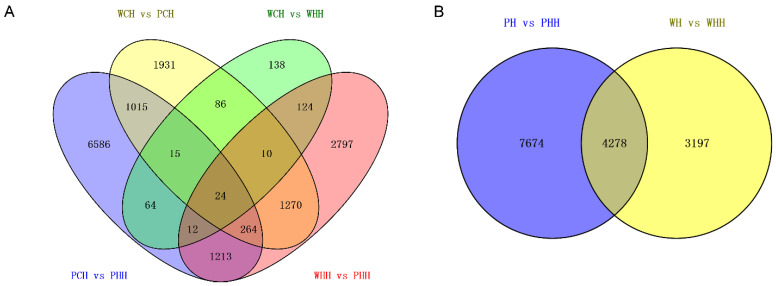
Venn plot of differentially expressed genes across different periods. (**A**) Adult stage. (**B**) Joint analysis of seedling and adult stages.

Only 24 shared DEGs were found at the adult plant stage, but 6586 genes were unique to *PLC* after heat treatment (Figure 3). Similarly, there were more DEGs unique to WHH vs. PHH, suggesting that *PLC* may have had a greater impact at the adult plant stage. We found a total of 4278 DEGs in the joint analysis and 7674 genes unique to PH vs. PHH after heat stress, which may also indicate that *PLC* had a greater impact on adult plants after heat stress (Figure 3).

After heat stress, there were differences in the number of some genes expressed in different genomes, so the gene data were analyzed to find the genomically insignificant differences in seedling and adult stages (Figure S1). WCH vs. PCH, WHH vs. PHH, and WC vs. PC had the highest number of genes in the B chromosome group, whereas all others had the highest number of genes in the D chromosome group (Figure S2). The differences between the seedling and adult stages of WT were evident in the B chromosome group. Our comparison revealed more genes involved in heat stress on chromosomes 2D, 2B, and 5D (Figure S1).

### 2.3. GO and KEGG Analyses

**Figure 4:**
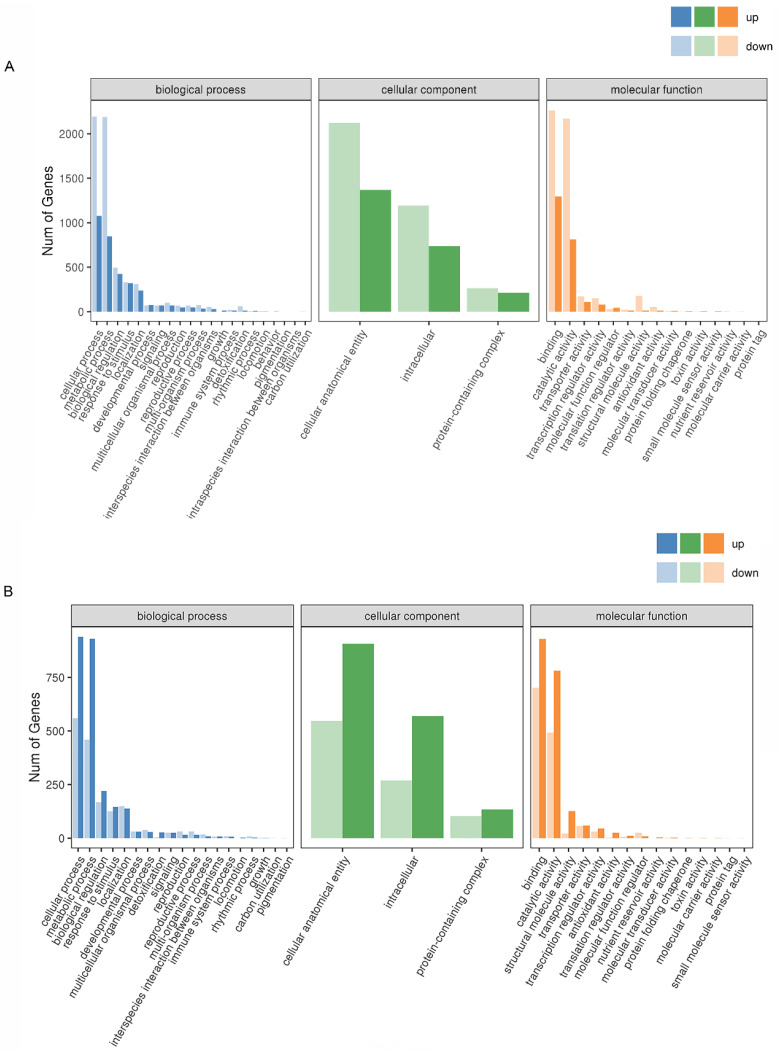
GO analysis of differentially expressed genes. (**A**) PCH vs. PHH. (**B**) WCH vs. PCH.

To further investigate the differences in wheat at different stages after heat stress and better understand the regulatory mechanism of the *TaPLC1-2B* gene, GO and KEGG enrichment analyses were conducted on DEGs at different stages. GO analysis on DEGs revealed no differences between the seedling and adult stages. In biological processes (BP), DEGs were mainly enriched in cellular processes, metabolic processes, and biological simulation pathways. Cellular components (CC) were mainly enriched in cellular anatomical entity, intracellular, and protein-containing complex pathways (Figure 4 and Figures S3 and S4). The DEGs in the molecular functions category were mainly enriched in the binding, catalytic activity, and transporter activity pathways. We conducted GO enrichment analysis on PH vs. PHH and WH vs. WHH, and found that the DEGs in the adult and seedling stages were related to cell membranes and organelles, while the CC enrichment in the seedling stage was mainly related to cellular and intracellular anatomical entities (Figure S5).

**Figure 5:**
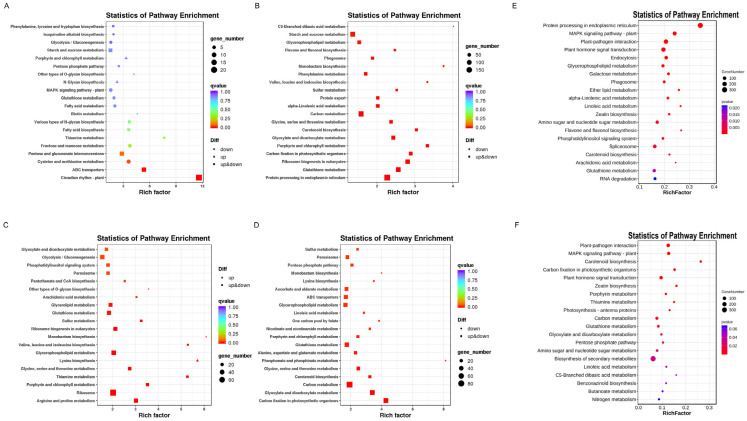
KEGG pathway significant enrichment analysis. (**A**) WCH vs. WHH. (**B**) PCH vs. PHH. (**C**) WCH vs. PCH. (**D**) WHH vs. PHH. (**E**) Enrichment analysis of DEGs after heat stress in wild-type seedlings. (**F**) Enrichment analysis of DEGs after heat stress in *PLC* seedlings. The figure shows the top 20 pathways with the lowest significant q-value.

KEGG analysis was also conducted on DEGs. After heat stress at the seedling stage, the KEGG of DEGs showed enrichment of the protein processing in the endoplasmic reticulum, the MAPK signaling pathway, plant–pathogen interaction, and the plant hormone signal transduction pathways (Figure 5E). After heat stress in the WT adult stage, KEGG identified enrichment mainly in the circadian rhythm plant and the ABC transporter pathways (Figure 5A). *PLC* led to the enrichment of the biosynthesis of secondary metabolites, plant–pathogen interaction, the MAPK signaling pathway, and plant hormone signal transduction in DEGs during the seedling stage (Figure 5F). During the adult stage, *PLC* was enriched in ribosome and glycerophospholipid metabolism (Figure 5C). After heat stress, *PLC* was enriched in protein processing in the endoplasmic reticulum, glutathione metabolism, and carbon metabolism during the adult stage (Figure 5B). These results indicated differences in responses to heat stress between the seedling and adult stages. To further understand these differences, we made a comparison of the PH vs. PHH and WH vs. WHH pathways. We found that *PLC* during the adult stage could trigger the glycerophospholipid metabolism, which was a pathway that did not exist in *PLC* and WT during the seedling stage (Figure S6). Glycine, serine, and threonine metabolism, as well as cysteine and methionine metabolism, are pathways that are unique to the adult stage and do not exist at the seedling stage. This indicated that different KEGG pathways were used during the seedling and adult stages to improve wheat’s heat tolerance, while *TaPLC1-2B* enhanced heat tolerance through the plant–pathogen interaction and MAPK signaling pathways during the seedling stage, and through a glycerol phospholipid metabolism pathway; glycine, serine, and threonine metabolism; and cysteine and methionine metabolism during the adult stage.

### 2.4. Analysis of DEGs for Plant Hormone Signaling

Hormonal signaling pathways for AUX, cytokinin (CTK), gibberellin (GA), abscisic acid (ABA), brassinosteroids (BR), jasmonic acid (JA), and salicylic acid (SA) in plants respond to abiotic stress. The number of genes for the different hormones was greater at the seedling stage than at the adult stage (Figure S7). AUX differed most markedly between the seedling and adult stages, and most of the genes were more highly expressed at the seedling stage than at the adult stage (Figure S7). However, certain genes exhibit increased expression levels during the adult stages as opposed to the seedling stages, for example, *TraesCS7D03G1098600*, an AUX-related gene. After heat stress, the levels of *TraesCS7D03G1098600* expression in the seedling stages of WT and *PLC* were 35.3 and 38.6, respectively, and there was no significant difference between WT and *PLC* (Table S2). However, the expression level of *TraesCS7D03G1098600* was 70.9 after heat stress during the adult stage of *PLC*. Through PC vs. PCH analyses, it was determined that the expression level of *TraesCS7D03G1098600* was higher in the adult stage than in the seedling stage (Table S3). The number of AUX upregulated genes was significantly higher than the number of downregulated genes in WH vs. WHH and PH vs. PHH, which indicated that there was a difference in AUX between the adult and seedling stages after heat stress (Table S4). The number of PH vs. PHH genes was greater than that of WH vs. WHH, suggesting that *PLC* is involved in different regulatory mechanisms at the adult and seedling stages. Both heat stress and *PLC* caused a downregulation in hormone expression, especially for AUX, and the differences were not significant for other hormones (Figure S7). The basal expression of ABA in the seedling stage is higher than in the adult stage, but the expression of some genes decreases rapidly after heat stress. However, the expression level of ABA in the adult stage rapidly increased after heat stress (Figure S8). Through heat maps, it was found that the expression level of GA-related genes in the adult stage was higher than in the seedling stage. *PLC* improves heat tolerance by increasing the expression of GA-related genes. However, the expression level of GA rapidly decreased after heat stress (Figure S8).

### 2.5. DEG Analysis of Heat Shock Proteins and Heat Shock Factors

Heat shock proteins (HSPs) and heat shock factors (HSFs) responded rapidly in wheat after heat stress, with more than 120 upregulated HSP genes and almost no downregulated genes expressed after heat stress in *PLC* and WT at the seedling stage (Table S5). The number of upregulated genes in HSFs after heat stress was greater than the number of downregulated genes in both *PLC* and WT at the seedling stage, and there were more downregulated genes in WT than in *PLC* (Table S6). The amount of HSPs and HSFs in *PLC* and WT at the adult stage was lower than that at the seedling stage after heat stress.

### 2.6. DEG Analysis of TFs

**Figure 6:**
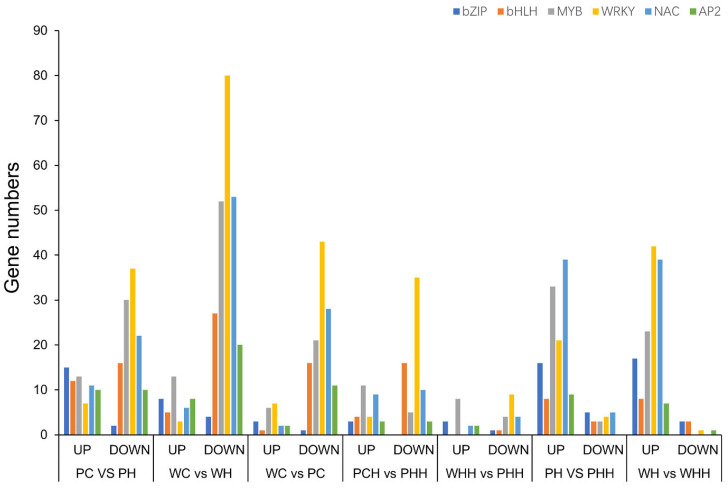
Analysis of transcription factors expressed differently at different stages.

There are complex and diverse TFs in plants, and we analyzed the differences in major TFs—such as *AP2*, no apical meristem (NAC), *bHLH*, *bZIP*, and *WRAK*—between the seedling and adult stages. After heat stress, it was revealed that the number of TFs in the seedling stage was greater than the number of TFs present in the adult stage. Further analysis in PH vs. PHH and WH vs. WHH showed that the number of upregulated genes was greater than the number of downregulated genes, which was opposite to the majority of downregulated genes in the seedling and adult stages. However, in WT there were no significant differences in *bHLH*, *AP2*, and *bZIP* between the seedling and adult stages. After heat treatment, the downregulated *MYB* genes in the adult stage of *PLC* were at a much smaller number than in the seedling stage (Figure 6). The number of upregulated genes in *bZIP*, *bHLH*, and *AP2* after *PLC* heat stress in the seedling stage was higher than those found in the adult stage, while the number of downregulated genes in *MYB* during the adult stage was lower than that of the seedling stage. This indicates that there were differences in the regulation of TFs between the seedling and adult stages after heat stress.

## 3. Discussion

RNA-seq can be used to analyze the mechanisms of drought, salt, and cold tolerance, and also to uncover the role of lncRNA and the identification of candidate genes [23,24,26,28,29,30]. In this study, RNA-seq was used to analyze the differences in mechanisms between the seedling and adult stages after heat stress. We found that the number of downregulated genes was greater than the number of upregulated genes after heat stress at both the seedling and adult plant stages. Previous research has shown that the number of downregulated genes was greater than the number of upregulated genes after heat stress in wheat seeds, and the number of downregulated genes was also greater than the number of upregulated genes after heat stress in *Brachypodium distachyon* [31,32]. This is consistent with our findings. We concluded that the adult plant stage may be protected against heat stress through the upregulation of gene expression, while the seedling stage had the opposite response. After heat stress, we analyzed the situation of DEGs and found that the number of upregulated genes was greater than the number of downregulated genes in PH vs. PHH and WH vs. WHH (Figure 2). Zhang et al. [27] previously found that there are different response mechanisms between the vegetative and reproductive tissues of *Arabidopsis* after heat stress, which also proved our viewpoint.

To further uncover the differences between the seedling and adult stages, we analyzed the chromosomal distribution of DEGs. Saidi et al. [26] found that DEGs on chromosomes were uniformly distributed, whereas we found that there were differences, although not significant, in the distribution of DEGs across chromosomes. Interestingly, both the seedling and adult stages of *PLC* had a higher number of B chromosomes, while the number of D chromosomes was higher after heat stress (Figure S1). According to reports, stress-related genes are preferentially preserved in allopolyploids, which is consistent with the number of heat stress-related genes found on the D chromosome [33]. Using recombinant inbred lines (RIL) constructed from the heat-tolerant variety “Halberd” and the heat-sensitive variety “Cutter”, heat stress-related quantitative trait loci (QTLs) were found on chromosomes 2A, 2B, and 1A [34]. Barakat et al. [35] found that JD_c4438_839, located on 5D, is a stable heat stress-related QTL. We also found that 2D, 2B, and 5D chromosomes were associated with heat stress.

To further explore the mechanisms of thermal responses during the adult and seedling stages, GO and KEGG analyses were conducted on DEGs. Previous studies have found that there are differences in carbohydrate metabolism between tolerant and intolerant materials after heat stress [36]. However, we found no significant difference between the seedling and adult stages in GO analysis. KEGG analysis found that after heat stress in the seedling stage, downstream genes and heat response genes were activated by regulating hormone signaling, MAPK signaling pathways, and plant–pathogen interaction pathways (Figure 5). A significant difference occurred in the adult stage during heat stress, which mainly improves the heat tolerance of wheat by activating heat responsive genes through carbon metabolism, circadian rhythm, glutathione metabolism, and ABC transport pathways (Figure 5). More transcriptome analyses after heat stress have found that metabolic pathways, carbon metabolism, and plant–pathogen interaction pathways were significantly enriched, which is consistent with our findings [25,37]. *TaPLC1-2B* enhances basal heat tolerance by responding to different pathways during the seedling and adult stages. *PLC* mediates the plant–pathogen interaction pathways and resistance-related genes in order to enhance basal heat tolerance during the seedling stage. We speculate that inducing pathway-associated molecular patterns (PAMPs) or effector-triggered immunity (ETI) during the seedling stage triggered an immunity enhancement in overall tolerance [38]. During the adult stage, *PLC* was involved in the ribosome and glycerophospholipid metabolism, and we speculate that it enhanced basal heat tolerance through lipid signaling [12,39]. Previous studies found a significant enrichment in glycine, serine, and threonine metabolism, along with cysteine and methionine metabolism, in *Sargassum pallidum* after heat stress [40]. However, we found that the glycine, serine, and threonine metabolism pathway and the cysteine and methionine metabolism pathway were not present at the seedling stage. In summary, these analyses demonstrated the clear differences in the mechanisms of heat stress response during the seedling and adult stages.

Phytohormones can mitigate plant heat damage in addition to playing roles in plant growth and development [41,42,43]. Results from WH vs. WHH and PH vs. PHH indicated that the adult stage upregulated the AUX gene in order to resist heat stress. However, the seedling stage increased heat tolerance through negative regulatory genes (Figure S7). Previous studies found that most AUX genes are downregulated after heat stress, which is consistent with our findings [44]. According to reports, AUX is necessary for plant morphogenesis under heat stress conditions, and heat stress during the seedling stage can lead to an increase in AUX concentration [45,46]. The overall expression level during the seedling stage exceeds that of the adult stage. We hypothesize that this is likely a result of the increased demand for auxin during the seedling stage to promote growth post heat stress. However, we found that *TraesCS7D03G1098600* gene are specifically upregulated after heat stress in adulthood, and these upregulated genes may be involved in the morphogenesis of adult plants.

In addition to acting as molecular chaperones, heat stress proteins are also involved in signaling pathways. For example, heat shock protein 90 (Hsp90) can be involved in growth hormone regulation, while other small heat stress proteins prevent protein aggregation by binding to proteins, and others can reduce the accumulation of reactive oxygen species (ROS) during stress [47,48,49]. After heat stress, HSPs and HSFs rapidly respond during the seedling and adult stages (Figures S9 and S10). In the present study, the number of upregulated genes in the seedling stage was greater than that in the adult stage, and we speculate that during the seedling stage the leaves were more sensitive to heat stress. Zhou et al. [50] found that the seedling and flowering stages of tomatoes are more suitable for identifying heat stress, which is consistent with our finding. According to reports, some HSFs exhibit reverse transcription under heat stress [44]. We also found that *PLC* could cause HSFs to downregulate genes in order to improve heat tolerance.

**Figure 7:**
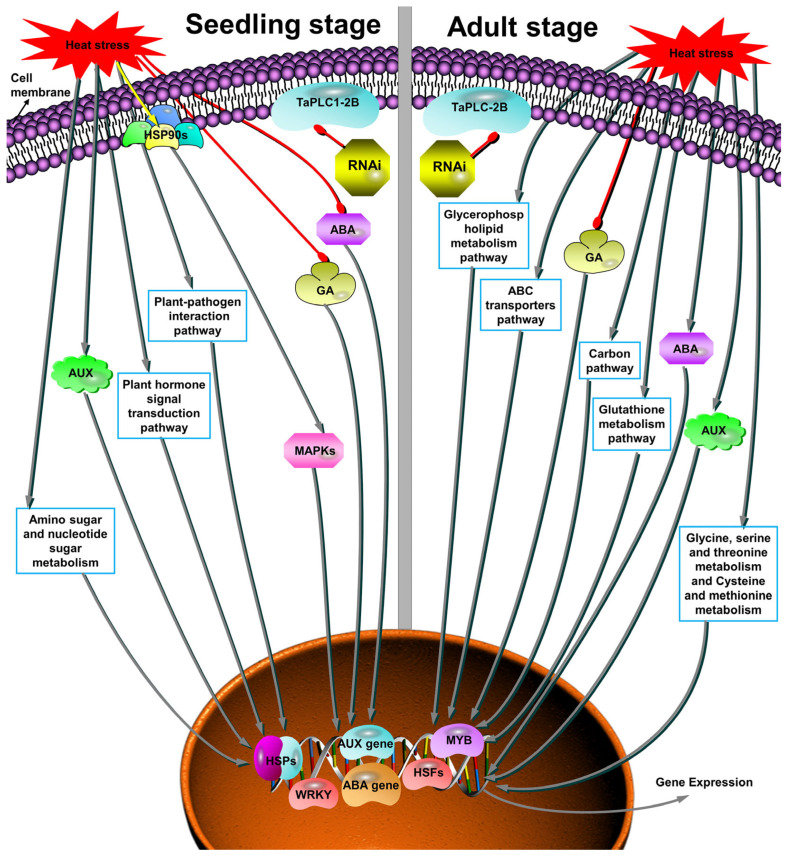
Pathway diagram of the participation in heat stress response during seedling and adult stages (Red lines represent the inhibition of gene expression. Gray lines represent the increase in gene expression).

In this study, it was found that major TF families—such as *bZIP*, *bHLH*, *MYB*, *WRKY*, *NAC*, and *AP2*—responded to heat stress, and it has been confirmed that these TFs are involved in the regulation of downstream genes after heat stress [51,52,53,54,55,56,57]. Previous studies have shown that the expression of *AtWRKY33* decreases under heat stress, but it works together with *AtWRKY25* and *AtWRKY26* to resist heat stress, and the overexpression of all three increases heat tolerance [54]. After heat stress in the seedling stage of WT, the *WRKY* gene is downregulated to resist heat stress (Figure 6). We speculate that this may have the same regulatory pattern as *AtWRKY33*. The *WRKY* gene is regulated through negative and positive regulation during the seedling and adult stages, respectively, to resist heat stress. However, after analyzing PH vs. PHH and WH vs. WHH, it was discovered that NAC-TFs exhibit a regulatory mechanism comparable to *WRKY*, with downregulation during heat stress in the seedling stage and upregulation during the adult stage (Figure 6). Previous studies have found that *OSPL* encodes a MYB family gene, the mutation of which enhances seed heat resistance, while another study found that overexpression of the *LlMYB305* gene in lilies enhances heat resistance [55,58]. In the present study, after heat stress in the WT seedling stage, MYB-related genes were downregulated, while WH vs. WHH and PH vs. PHH resisted heat stress by upregulating *MYB* genes. This indicated that negative regulation of *MYB* during the seedling stage increased heat tolerance, while positive regulation of *MYB* expression during the adult stage increased heat tolerance. However, we found that the expression patterns of MYB TFs in the seedling and adult stages of *PLC* are similar to those of WT. In summary, WRKY and MYB TFs exhibited differences in responses to heat stress during the seedling and adult stages. Heat tolerance was increased through negative regulation of genes during the seedling stage, while heat tolerance was increased through positive regulation during the adult stage (Figure 7).

## 4. Materials and Methods

### 4.1. Plant Material Growing and Heat Treatment

Kenong 199 (KN199) is a common wheat (*Triticum aestivum* L.) variety which was first bred in China in 2006. The KN199 material of *TaPLC1-2B* RNAi used in this study was created by Northwest A&F University, and the wild-type KN199 material was kept in the laboratory. Wheat seeds from both materials were first washed three times with sterilized distilled water, then had their surfaces sterilized with 75% alcohol for 5 min; after sterilization, the sterile distilled water was washed three times, then the surfaces of the seeds were sterilized with 5% sodium hypochlorite for 15 min, before being rinsed with sterile distilled water three times. The 9 cm quantitative filter paper (Double ring, Hangzhou, China) was placed in a 9 cm culture dish (Bkmam, Changde, Hunan, China) and 2 mL of sterile water was added. The seeds were then placed on moistened quantitative sterile filter paper for germination. Seeds that germinated consistently were picked and transplanted into individual pots (5.5 cm diameter, 6.0 cm deep), and three plants were planted in each pot. Wheat seedlings were cultivated in a low temperature vernalization incubator (Ningbo Jiangnan Instrument, Ningbo, China) at 4 °C for 4 weeks, with 16 h of light and 8 h of darkness during vernalization. After completion of vernalization, the samples were transplanted into pots (diameter 29.5 cm, depth 21.8 cm). The growth of wheat before vernalization was carried out in a greenhouse (Northwest A&F University, China); wheat was planted in the greenhouse after vernalization until harvest. The temperature of the greenhouse was 24 °C/22 °C (day/night), with 16 h of light and 8 h of darkness. The nutrient soil (Pindstrup, Ryomgaard, Denmark) utilized to transplant wheat seedlings had been sterilized.

For materials with uniform growth, sufficient samples are collected before heat treatment. When wheat reached the stage of two leaves and one heart, they were heat-treated at 40 °C for 30 min in a light incubator (Boxun, Shanghai, China), and 50 mL of water was poured into each pot two days before heat treatment. The samples were taken immediately after treatment and snap-frozen in liquid nitrogen for use in subsequent RNA extraction. Thirty days after vernalization, the wheat had reached the heading and flowering stages. Two days before the heat treatment, 400 mL of water was poured into each pot. During this period, wheat plants with uniform growth were selected and heat-treated at 40 °C for 30 min in a light incubator, then sampled immediately after treatment and snap-frozen in liquid nitrogen for use in subsequent RNA extraction. The collected samples were stored at −80 °C for the next transcriptome sequencing step.

### 4.2. RNA Extraction and Sequencing Analysis

Total RNA was extracted using a TransZol Plant kit (TransGen Biotech, Beijing, China), based on the manufacturer’s protocol. RNA from the desired samples was extracted using the RNA extraction kit, and then analyzed for RNA integrity using the RNA Nano 6000 assay kit (Agilent Technologies, Santa Clara, CA, USA) for the Agilent Bioanalyzer 2100 system (Agilent Technologies, CA, USA). Library preparation and sequencing were performed with BioMarker. Sequencing libraries were generated using the NEB Next Ultra^TM^ RNA Library Preparation Kit (NEB, Ipswich, MA, USA), according to the manufacturer’s recommendations, and the quality of the libraries was assessed on the Agilent Bioanalyzer 2100 system. After passing the assessment, the library preparations were sequenced using the Illumina Novaseq 6000 platform (Illumina, San Diego, CA, USA) and paired-end reads were generated.

### 4.3. RNA Sequencing Data Processing

Raw data in fastq format were processed using perl scripts, so as to facilitate the removal of splice-containing and low-quality reads from the raw data and obtain high-quality reads. After data processing, the reads were mapped to the reference genome sequence. The reference sequence for wheat is IWGSC RefSeq v2.1, downloaded from the IWGSC database (https://wheat-urgi.versailles.inra.fr/, accessed on 4 January 2023) [59]. Only reads with an exact match, or with one mismatch, are further analyzed and annotated against the reference genome. Mapping to the reference genome was performed using the Hisat2 2.1.0 software tool [60].

### 4.4. Identification of Differentially Expressed Heat-Responsive Genes (DEGs)

Genes from different samples were analyzed using DESeq2 1.42.0 [61], and Fold Change ≥ 2 and FDR < 0.01 were used as the screening criteria to obtain DEGs between samples.

### 4.5. GO and KEGG Enrichment Analysis

The DEGs were subjected to gene ontology (GO) enrichment analysis using the GOseq R package, and their functions were annotated [62]. KOBAS 3.0 is a python program that can contribute significantly to genome annotation. We used the KOBAS program to perform KEGG enrichment on the DEGs [63].

### 4.6. Transcription Factor Prediction

TF prediction of the obtained DEGs at seedling and adult stages was performed using the PlantTFDB database (http://planttfdb.gao-lab.org/, accessed on 14 September 2023) [64].

## 5. Conclusions

In this study, the seedling and adult plant stages of *TaPLC1-2B* RNAi-silenced wheat and WT were comparatively analyzed using RNA-seq technology. GO analysis of the differential genes showed that there were no differences in the pathways enriched between the seedling and adult stages. KEGG enrichment analysis revealed that the mechanisms involved in heat stress were different between the seedling and adult stages, with the seedling stage participating in the heat response mainly through hormone signaling and the MAPK pathway, among others, and the adult stage participating in the heat stress response through the pathway of carbon metabolism, among others. Moreover, *TaPLC1-2B* was also involved in different mechanisms at the seedling and adult plant stages. Heat tolerance was enhanced by increasing overall tolerance through the MAPK pathway and plant–pathogen interaction pathways during the *PLC* seedling stage, whereas heat tolerance was enhanced by activating downstream gene expression through lipid metabolism during the adult plant stage. Overall, our study has deepened our understanding of the mechanisms of heat tolerance in both the seedling and adult stages of wheat under transient heat treatment conditions.

## Data Availability

All data are available from the corresponding author upon reasonable request.

## Supplementary Materials

The supporting information can be downloaded at: https://www.mdpi.com/article/10.3390/ijms242316583/s1.
