# Supplementary material for: Exploring the Role of TaPLC1-2B in Heat Tolerance at Seedling and Adult Stages of Wheat through Transcriptome Analysis
Source: Int J Mol Sci. 2023 Nov 21;24(23):16583. doi: 10.3390/ijms242316583 (PMC10706844; doi:10.3390/ijms242316583)
Supplement: Supplementary file 1 [file ijms-24-16583-s001.zip › Supplementary Figures S1-S10.pdf]

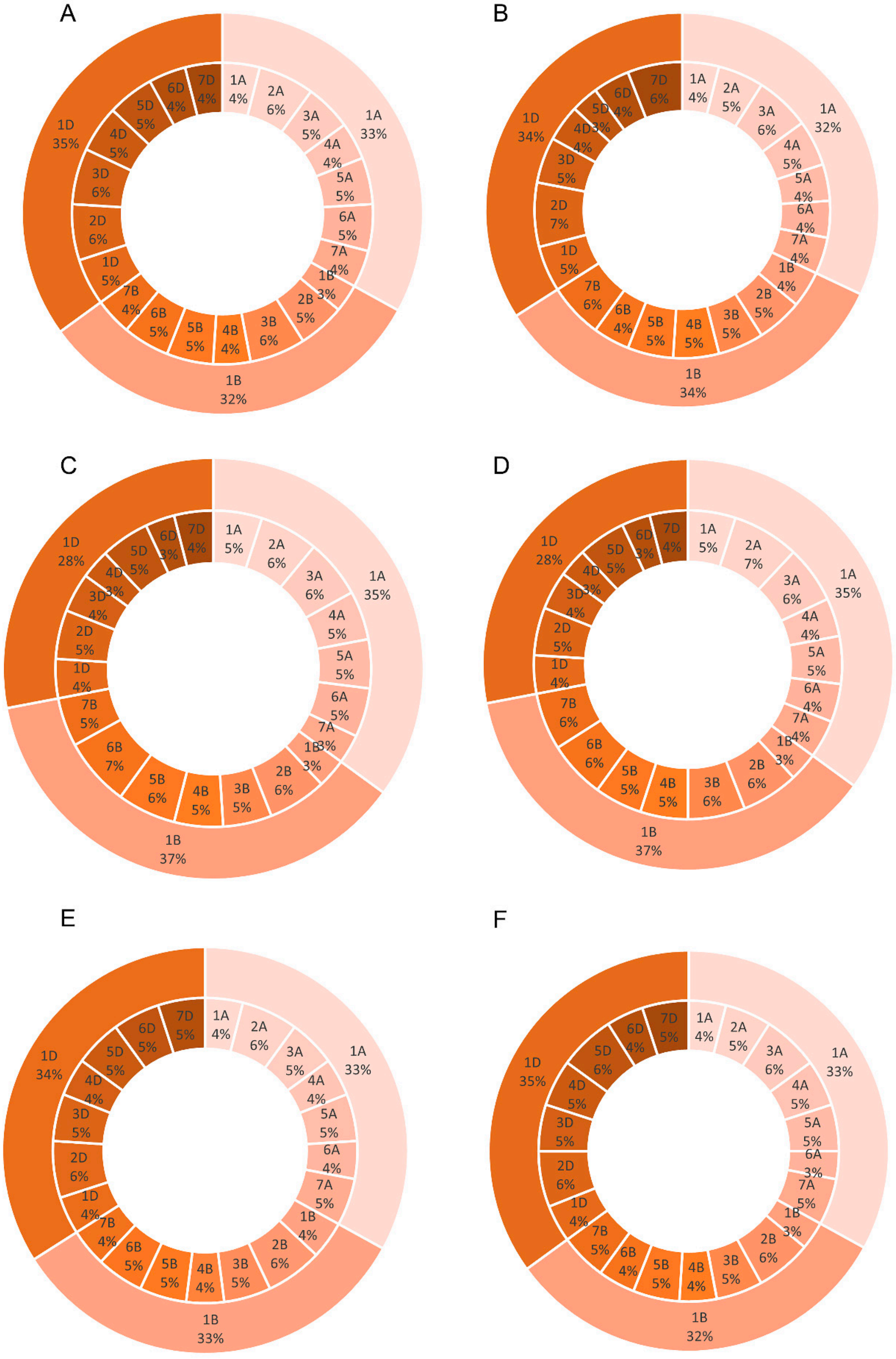

Figure S1. The distribution of differentially expressed genes on chromosomes. (**A**) DEGs of PCH vs. PHH. (**B**) DEGs of WCH vs. WHH. (**C**) DEGs of WCH vs. PCH. (**D**) DEGs of WHH vs. PHH. (**E**) DEGs of PH vs. PHH. (**F**) DEGs of WH vs. WHH.

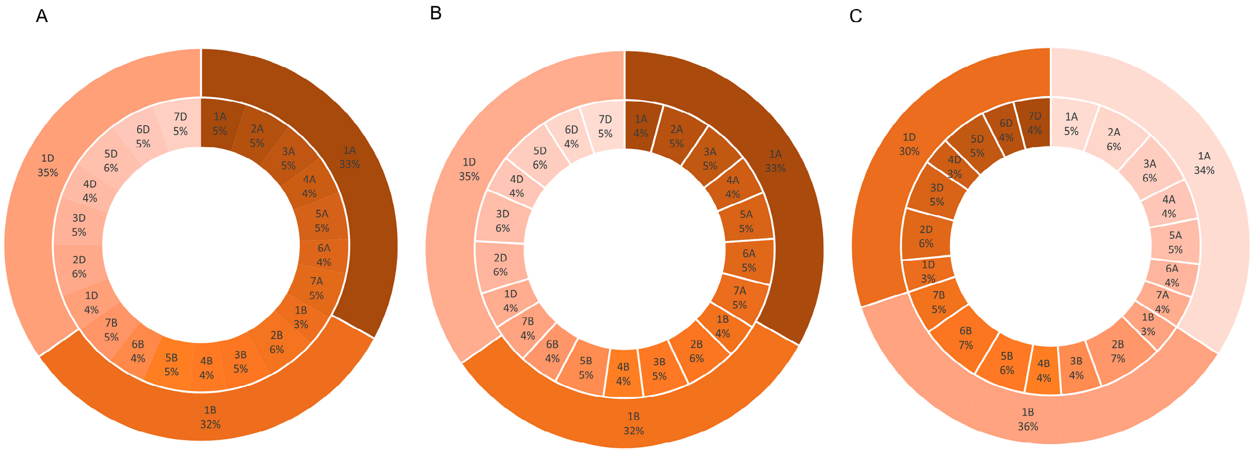

Figure S2. The distribution of differential genes on chromosomes. (**A**) DEGs of PC vs. PH. (**B**) DEGs of WC vs. WH. (**C**) DEGs of WC vs. PC.

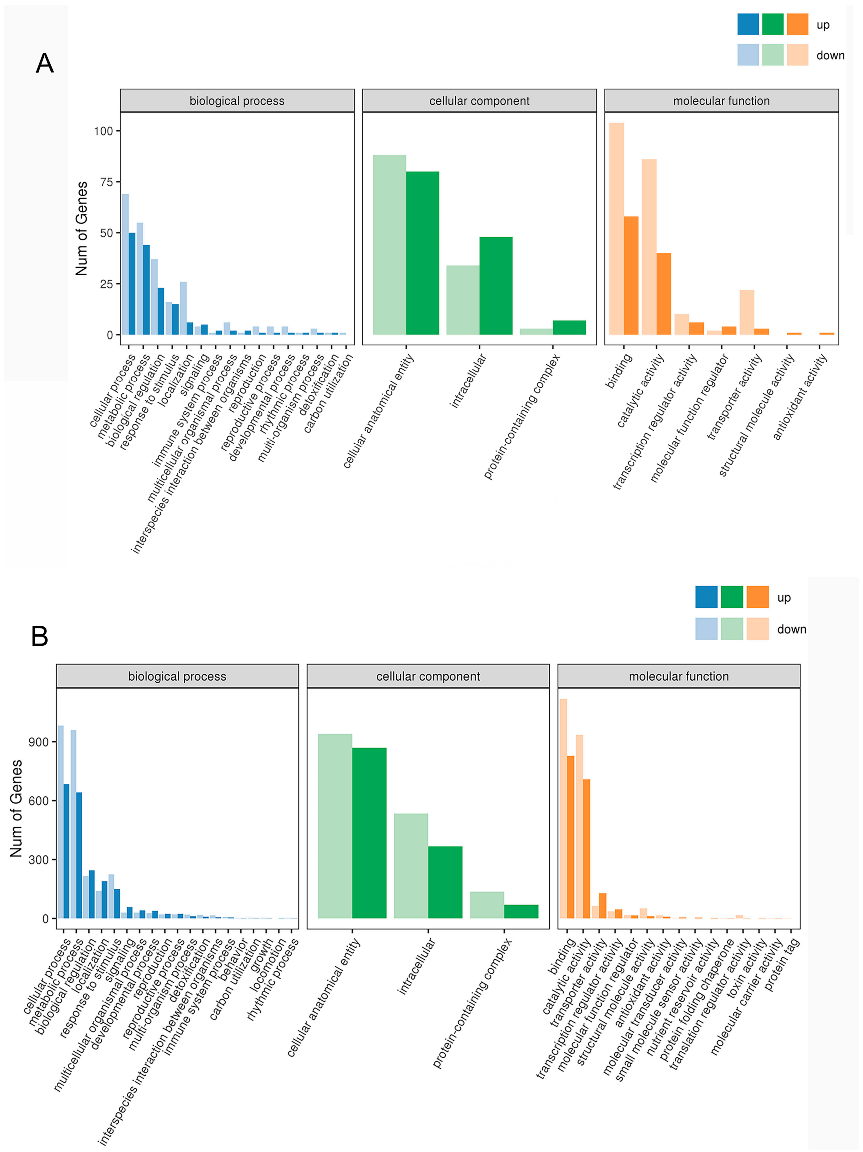

Figure S3. GO analysis of differentially expressed genes. (**A**) WCH vs. WHH. (**B**) WHH vs. PHH.

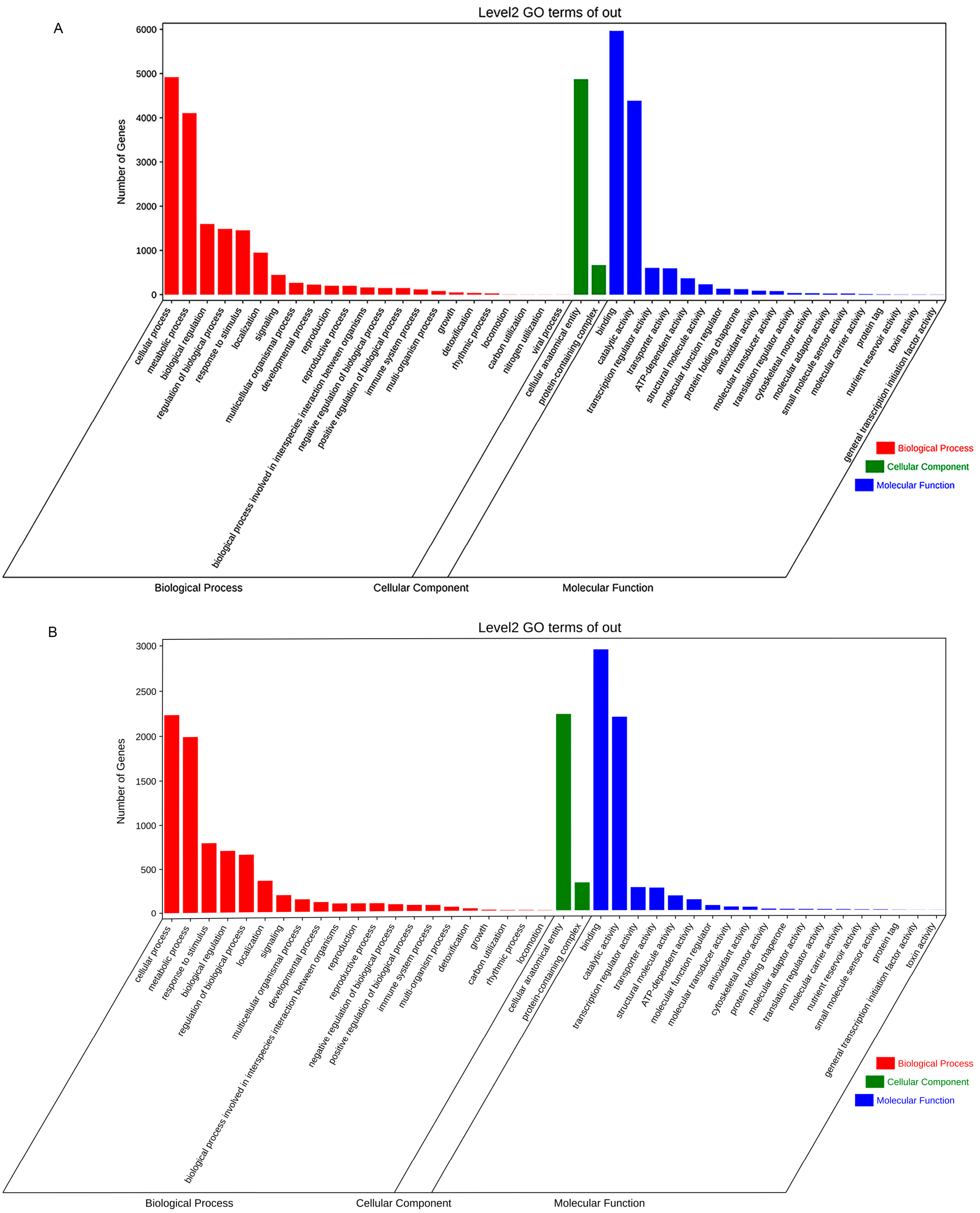

Figure S4. GO analysis of differentially expressed genes. (**A**) Heat stress treatment during seedling stage. (**B**) *PLC* heat stress treatment during seedling stage.

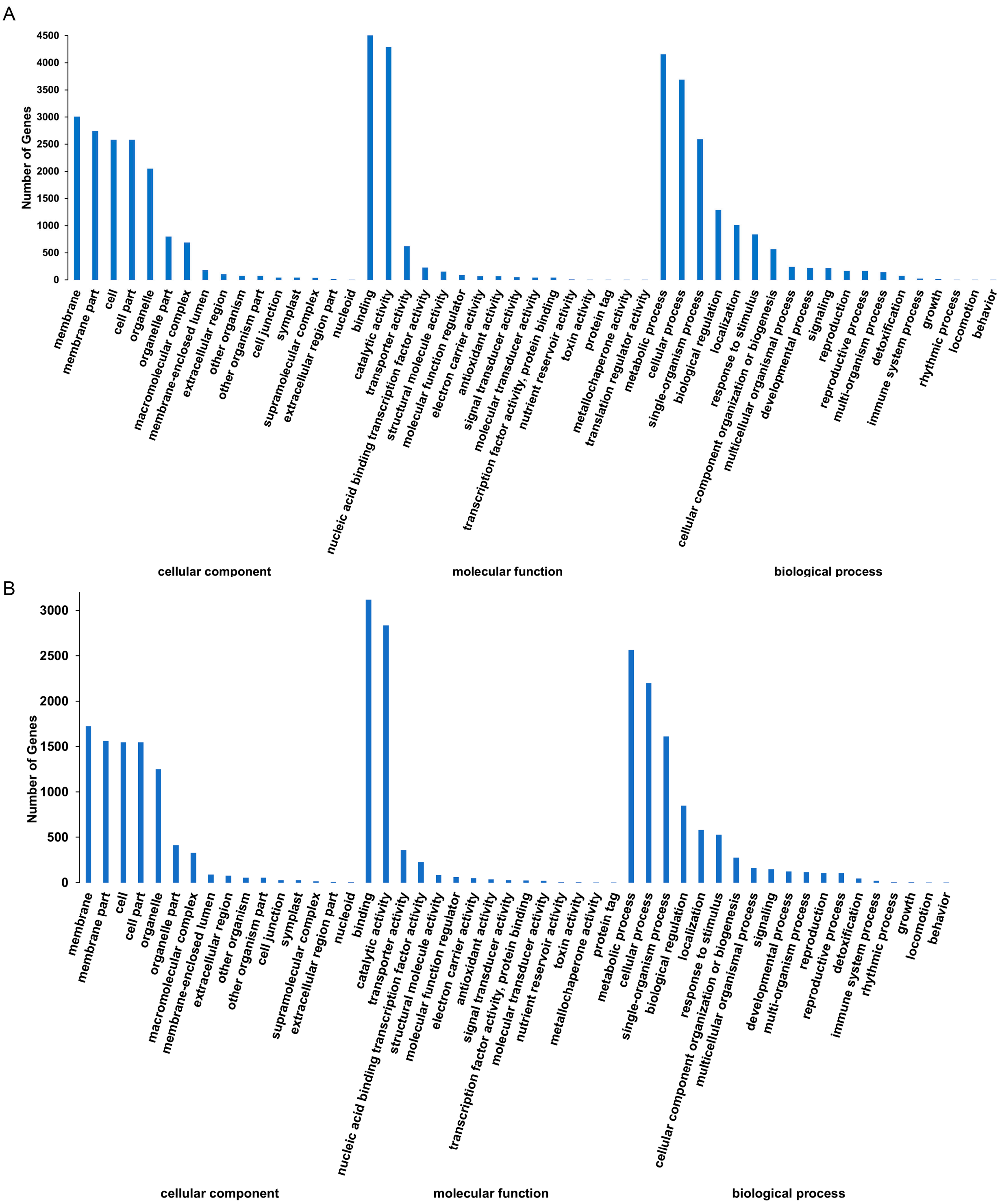

Figure S5. GO analysis of differentially expressed genes. (**A**) PH vs. PHH. (**B**) WH vs. WHH.

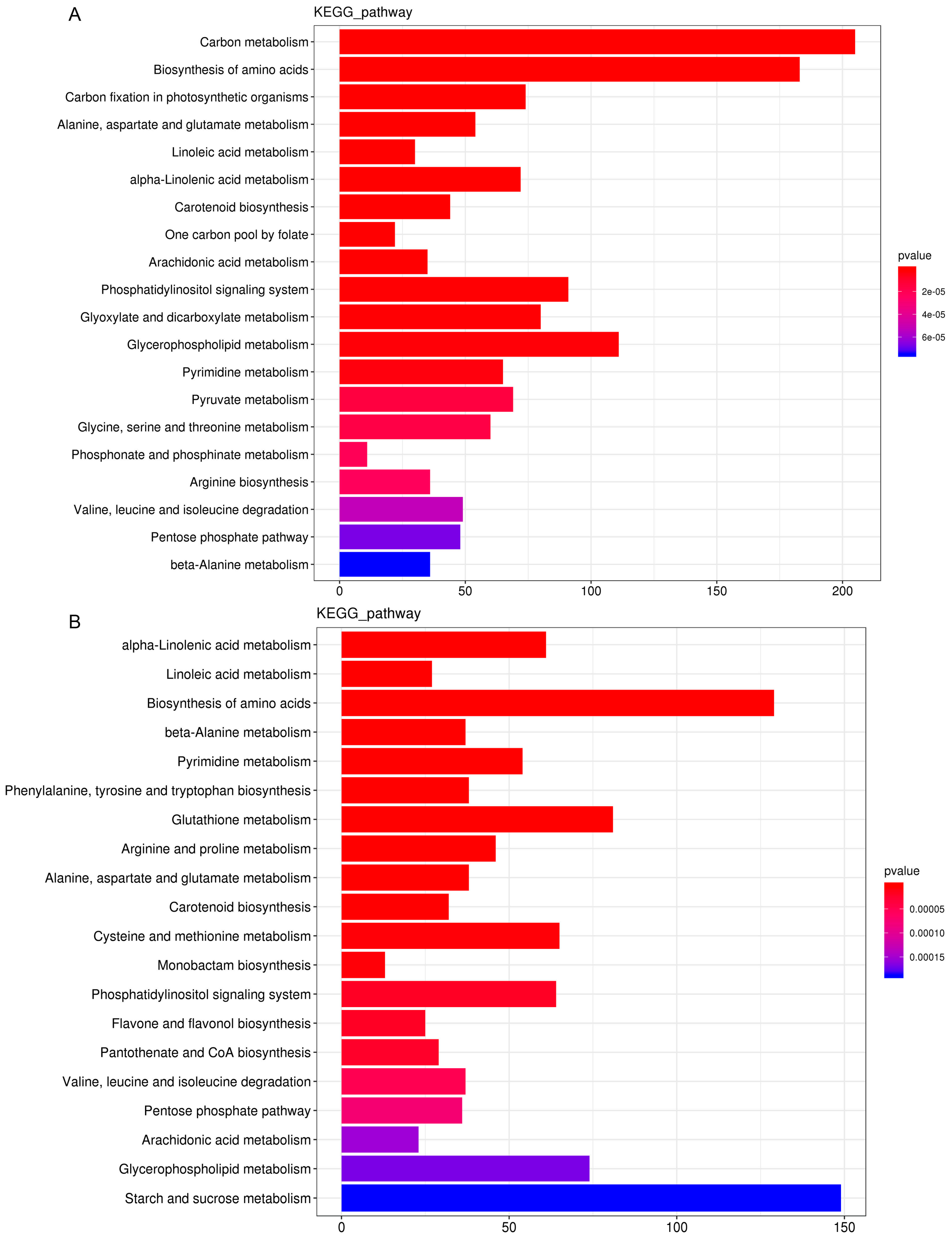

Figure S6. KEGG pathway significant enrichment analysis. (**A**) PH vs. PHH. (**B**) WH vs. WHH.

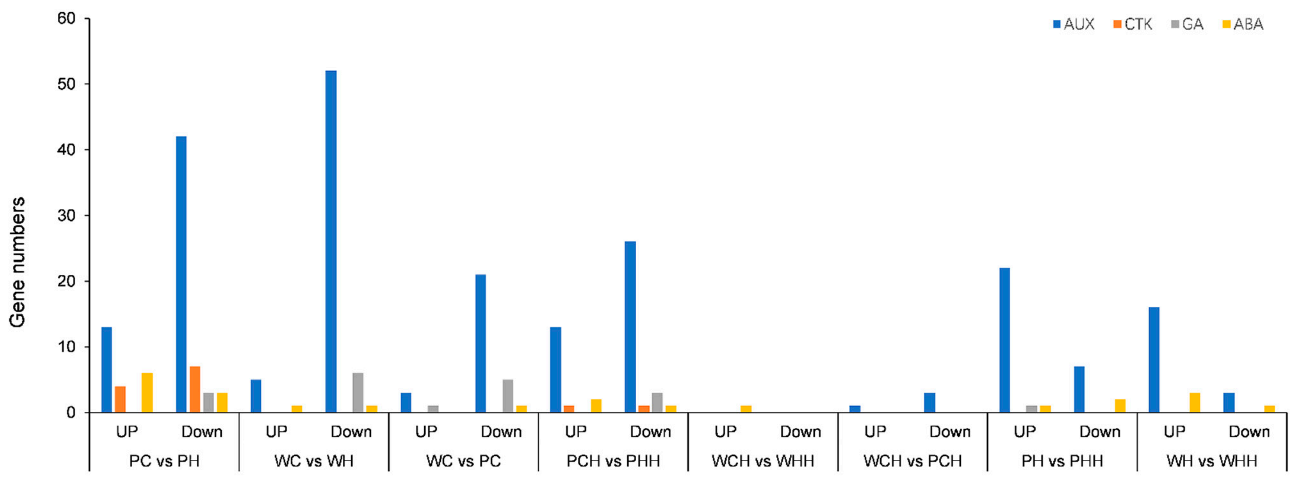

Figure S7. Statistical analysis of hormone DEGs.

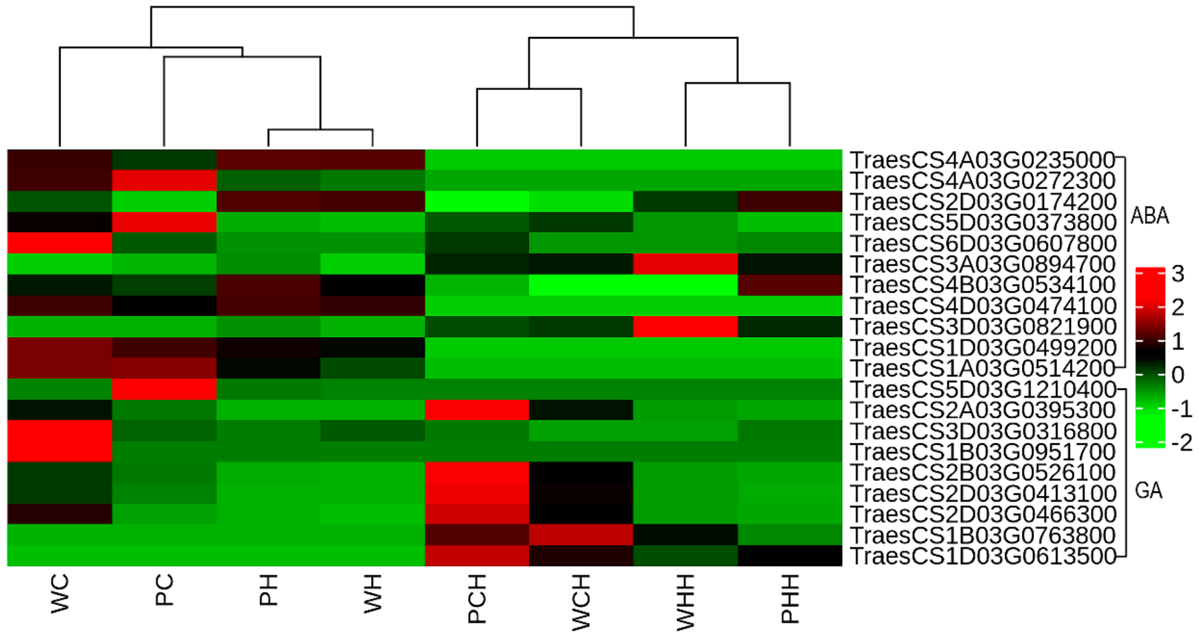

Figure S8. Heatmap analysis of the ABA and GA related genes between the control and the heat-stress treated.

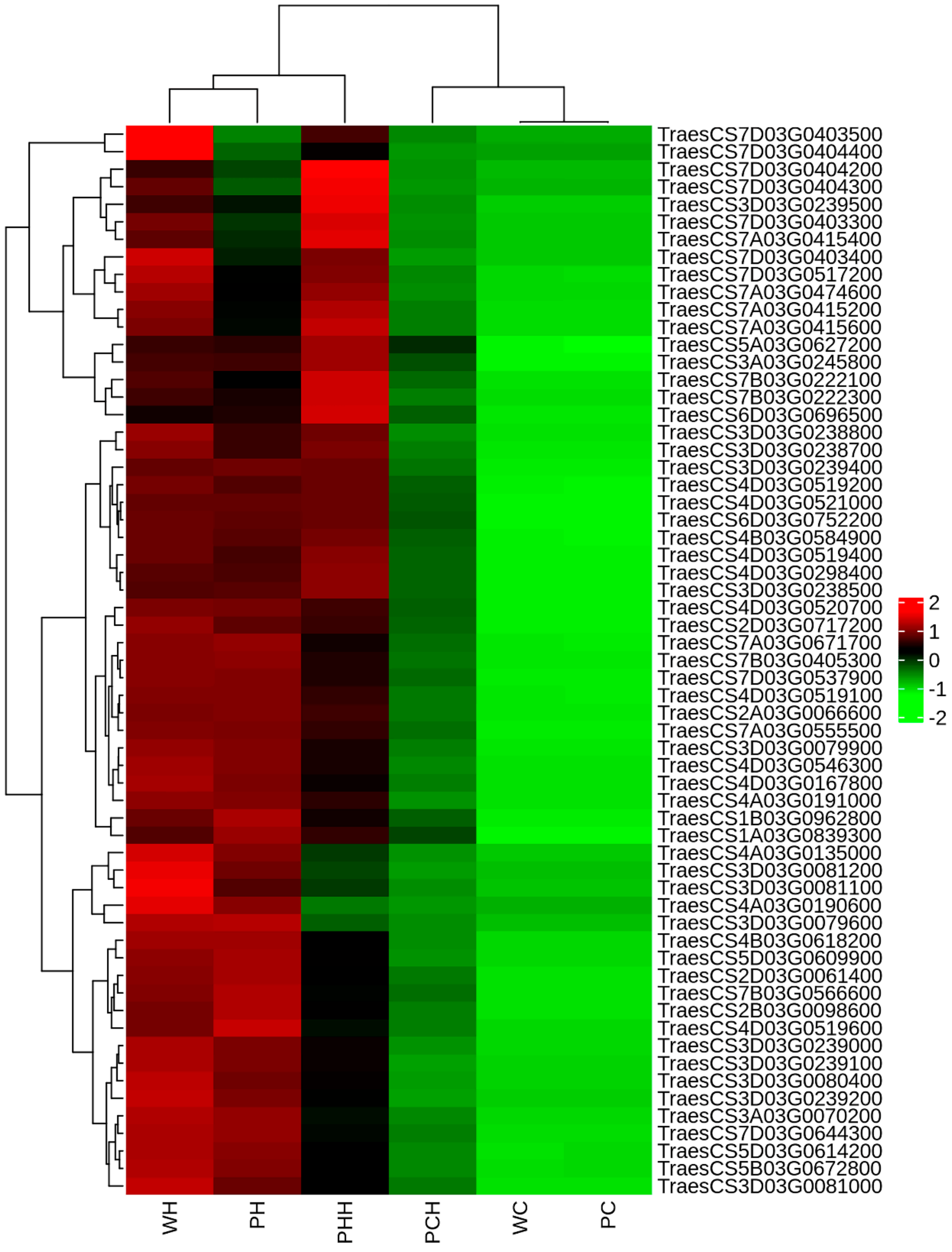

Figure S9. Heat map analysis of HSP genes shared by the control group and heat stress treatment group.

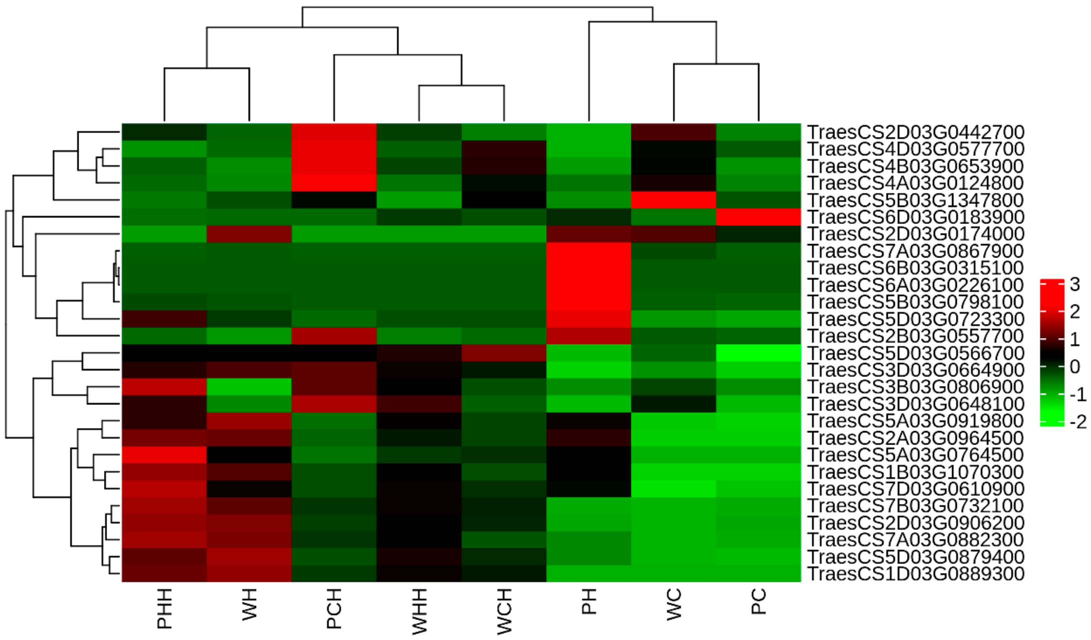

Figure S10. Heatmap analysis of the HSF gene between the control and the heat-stress treated.
